# Epidemiological patterns of animal bites in urban and rural areas of Sarakhs County, Iran: a cross-sectional study (2017–2024)

**DOI:** 10.1186/s12917-026-05638-x

**Published:** 2026-06-23

**Authors:** Elaheh Lael-monfared, Maryam Paydar, Sahar Mohammad Nabizadeh, Ehsan Mosa Farkhani, Maryam Mohammadi

**Affiliations:** 1 Social Determinants of Health Research Center, Basic Sciences Research Institute, Mashhad University of Medical Sciences, Mashhad, Iran; 2 Department of Health Education and Health Promotion, School of Health, Mashhad University of Medical Sciences, Mashhad, Iran; 3 Department of Environmental Health Engineering, School of Health, Mashhad University of Medical Sciences, Mashhad, Iran; 4 Department of Environmental Civil Engineering, Shahrood University of Technology-Iran, Shahrood, Iran; 5 Department of Epidemiology, School of Health, Social Determinants of Health Research Center, Mashhad University of Medical Sciences, Mashhad, Iran

**Keywords:** Animal bites, Epidemiological, Public health, Rural population, Cross-sectional study, Iran

## Abstract

**Background:**

Animal bites are a significant public health concern, particularly in regions with high exposure to domestic and wild animals. Understanding their epidemiological patterns and determinants is essential for effective rabies prevention and control. This study aimed to investigate the epidemiological patterns and determinants of animal bites in urban and rural areas of Sarakhs County, Iran, from 2017 to 2024.

**Methods:**

This cross-sectional descriptive-analytical study analyzed 6,371 recorded cases of animal bites reported to rabies prevention centers in Sarakhs County from 2017 to 2019. Data were collected using a census approach and extracted from the national portal system. Demographic and clinical variables were analyzed using SPSS v.26. Descriptive statistics and chi-square tests were employed to identify significant associations.

**Results:**

The average annual incidence rate was 790.12 per 100,000 population. The majority of bite victims were male (78.8%), and most incidents occurred in the 20–49 age group (41.5%). Students constituted the most affected occupational group (28.1%). A large proportion of bites occurred in rural areas (84.4%), mainly during the spring season (27.2%), with a peak in May (9.5%). Dog bites accounted for 90.1% of all cases, and 95% of the biting animals were domestic. The most common site of injury was the lower limbs (57.3%), and in 43.2% of cases, the bites occurred in the afternoon hours. A statistically significant relationship was observed between the frequency of bites and variables such as age, sex, occupation, time of day, season, type of animal, and injury site (*p* < 0.001).

**Conclusion:**

Animal bites in Sarakhs County predominantly affect rural male students and are largely caused by domestic dogs. The findings highlight the need for targeted educational programs and preventive measures, especially in rural communities and among school-aged populations.

## What is already known on this topic?

- ▪ Animal bites, particularly from domestic animals, pose significant public health risks and are associated with rabies transmission.
- ▪ Previous studies have indicated that certain demographics, such as age and gender, are more vulnerable to animal bites, often in rural settings.
- ▪ There is a recognized seasonal variation in animal bite incidents, with peaks often occurring during specific months.

## What this study adds

- ▪ This study provides comprehensive data on 6,371 animal bite cases in Sarakhs County, highlighting specific demographic trends and seasonal patterns.
- ▪ It emphasizes that dog bites account for a vast majority (90.1%) of cases, primarily affecting rural male students during the spring months.
- ▪ The study identifies statistically significant relationships between bite frequency and various factors, including age, sex, occupation, and time of day.

## How this study might affect research, practice, or policy

- ▪ Findings can inform the development of targeted educational programs and preventive measures aimed at high-risk groups, particularly in rural areas.
- ▪ The study underscores the need for policies addressing domestic animal control and public awareness campaigns about animal bite prevention.
- ▪ It opens avenues for further research into the effectiveness of prevention strategies and the socio-economic factors influencing animal bite incidents.

## Introduction

Rabies is a viral disease that can be transmitted between humans and animals, posing a serious public health threat. It is one of the oldest and most significant zoonotic diseases, primarily due to its high mortality rate of nearly 100% and the economic burden it creates [1]. Most animal bite injuries are caused by dogs and cats, which account for the majority of reported bite cases worldwide. Other domestic and wild animals contribute to a smaller proportion of cases [2]. These bites can manifest as punctures, abrasions, or lacerations. Typically, animal bites occur as a result of instinctive behavior when the animal feels threatened or is attempting to obtain food [3]. These bites can lead to infections in both humans and other animals. Rabies, in particular, is one of the most dangerous viral diseases transmitted this way, affecting all mammals, including livestock [4].

Animal bites present a major public health challenge due to their immediate health risks and the potential economic burden they impose on countries. More than 2.5 million people worldwide are exposed to animal bites and related injuries each year, which may lead to serious complications, including infection and, in some cases, death. Rabies remains a major public health concern, particularly in endemic regions [5].

Beyond the financial costs associated with preventing and treating these incidents, the psychological, social, and lasting effects of animal bites can significantly impact the lives of both the individuals affected and their families [6].

Animal bites, which often occur during interactions with pets, livestock, or wild animals, pose a risk of rabies virus transmission, especially in regions where rabies is common [7]. Dogs are responsible for the majority of human and animal bite injuries, accounting for over 90% of cases, followed by cats, humans, and rodents [7, 8]. In low-income countries, dogs are the primary source of these bites, contributing to increased rabies prevalence and higher mortality rates. This situation is further aggravated by limited access to post-exposure anti-rabies treatment [9]. The global mortality associated with canine rabies is estimated at approximately 59,000 deaths annually, with the highest burden occurring in Asia and Africa, particularly in rural areas. The World Health Organization (WHO) reports that most of these deaths (about 59.6%) occur in Asian and African countries, largely due to limited access to healthcare services and inadequate health systems [10].

Animal bites remain a major public health concern worldwide, particularly in regions with high exposure to domestic and wild animals. Rabies is responsible for a substantial burden of disease, with more than 99% of disability-adjusted life years lost attributed to premature death. In addition, the economic costs associated with rabies vary considerably across different regions of the world [11]. Globally, more than 10 million animal bite cases are reported annually, with the majority occurring in Asia and Africa, where dogs represent the primary source of exposure [12].

In Iran, rabies continues to impose a significant economic and health burden, with approximately 12 million USD spent annually on rabies vaccines. Although effective post-exposure prophylaxis is available, vaccination remains the only life-saving intervention after animal bites, as rabies is almost invariably fatal once clinical symptoms appear. Over the past two decades, both animal rabies and human exposure cases have increased in the country. For instance, reported animal bite cases increased from 23,625 cases (incidence rate: 56 per 100,000 population) in 1989 to 386,521 cases (incidence rate: 453 per 100,000 population) in 2023. This increase is partly attributed to improved surveillance systems and enhanced reporting, which have also contributed to a reduction in the ratio of human rabies deaths to animal bite incidence—an important indicator of effective post-exposure management [13]. Iran is among the countries where rabies is endemic in both wild and domestic animals, making it a continuing public health challenge due to its high fatality rate, increasing human exposure, livestock losses, and associated economic burden [14].

Khorasan Razavi Province, the largest province in Iran, covers approximately 20% of the country’s total area. Sarakhs County, located in the northeastern part of the province and bordering Turkmenistan, includes several rural and border communities with unique ecological and socio-demographic characteristics that may influence exposure to animal bites. Given its geographical position and cross-border interactions, understanding the local epidemiology of animal bites is essential for effective prevention and control strategies. Therefore, this study aimed to investigate the epidemiological patterns and determinants of animal bites in urban and rural areas of Sarakhs County, northeastern Iran, from 2017 to 2024, using data from the national rabies prevention registry.

## Methods

### Study site and population

**Fig. 1:**
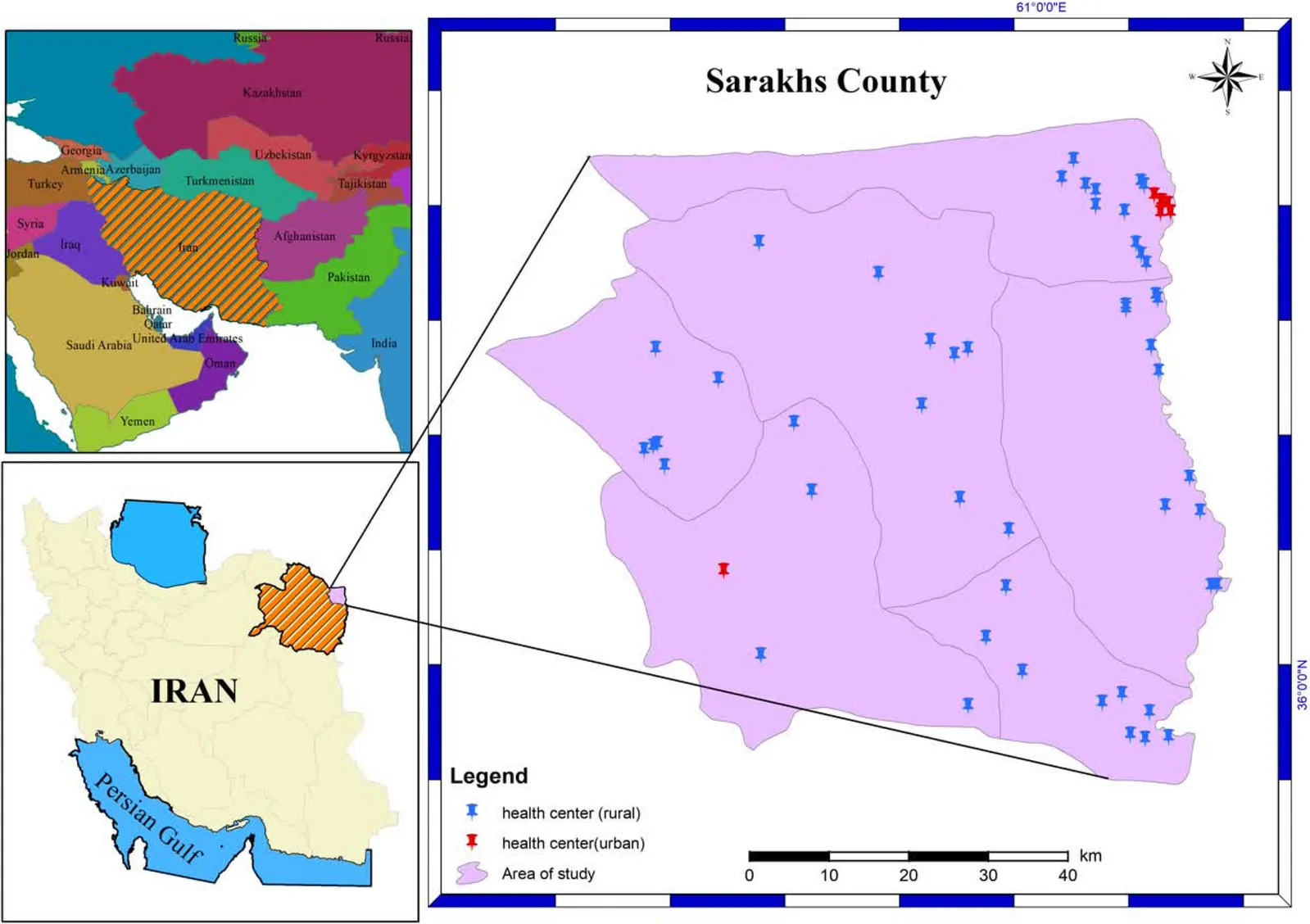
Geographical location of Iran: Khorasan Razavi Province, Sarakhs County

This study was conducted in Sarakhs County, located in northeastern Khorasan Razavi Province, Iran, covering an area of about 6,011 km². Sarakhs borders Turkmenistan to the north and east, and Mashhad to the southwest [15] (Fig. 1). The study population included all individuals referred to the county’s health center due to animal bites. Data were extracted from the National Rabies Prevention Registry, which routinely collects information such as age, sex, occupation, residence area, bite characteristics, and preventive measures.

### Study design

This cross-sectional descriptive-analytical study analyzed 6,371 animal bite cases reported to rabies prevention centers in Sarakhs from 2017 to 2024. All recorded cases were included using a census approach. Data were based on factors like demographic details, injury site, and type of animal, location, and treatment. Cases with incomplete records or those only visiting for follow-up vaccine doses were excluded.

### Data management

Data were extracted from the national portal, organized in Excel, and reviewed by provincial experts. Missing data were completed using registration office records. Final data were analyzed using SPSS version 26, employing descriptive and chi-square statistical tests.

Data were extracted from the Sina electronic national rabies prevention registry (Sina system), which is a standardized surveillance platform used in Iran for recording animal bite cases and post-exposure prophylaxis services. Data entry is performed routinely by trained healthcare staff at rabies prevention centers. Variables included demographic characteristics (age, sex, occupation, residence), injury-related factors (bite site, severity, number of wounds), animal-related information (type, ownership status), and temporal distribution of cases.

### Inclusion/exclusion

All registered animal bite cases from 2017 to 2024 were included. Cases with incomplete key variables or duplicate follow-up vaccination records were excluded.

### Ethics

Ethical approval for this study was obtained from the Ethics Committee of Mashhad University of Medical Sciences (IR.MUMS.REC.1403.008). As secondary anonymized registry data were used, informed consent was waived.

## Result

**Fig. 2:**
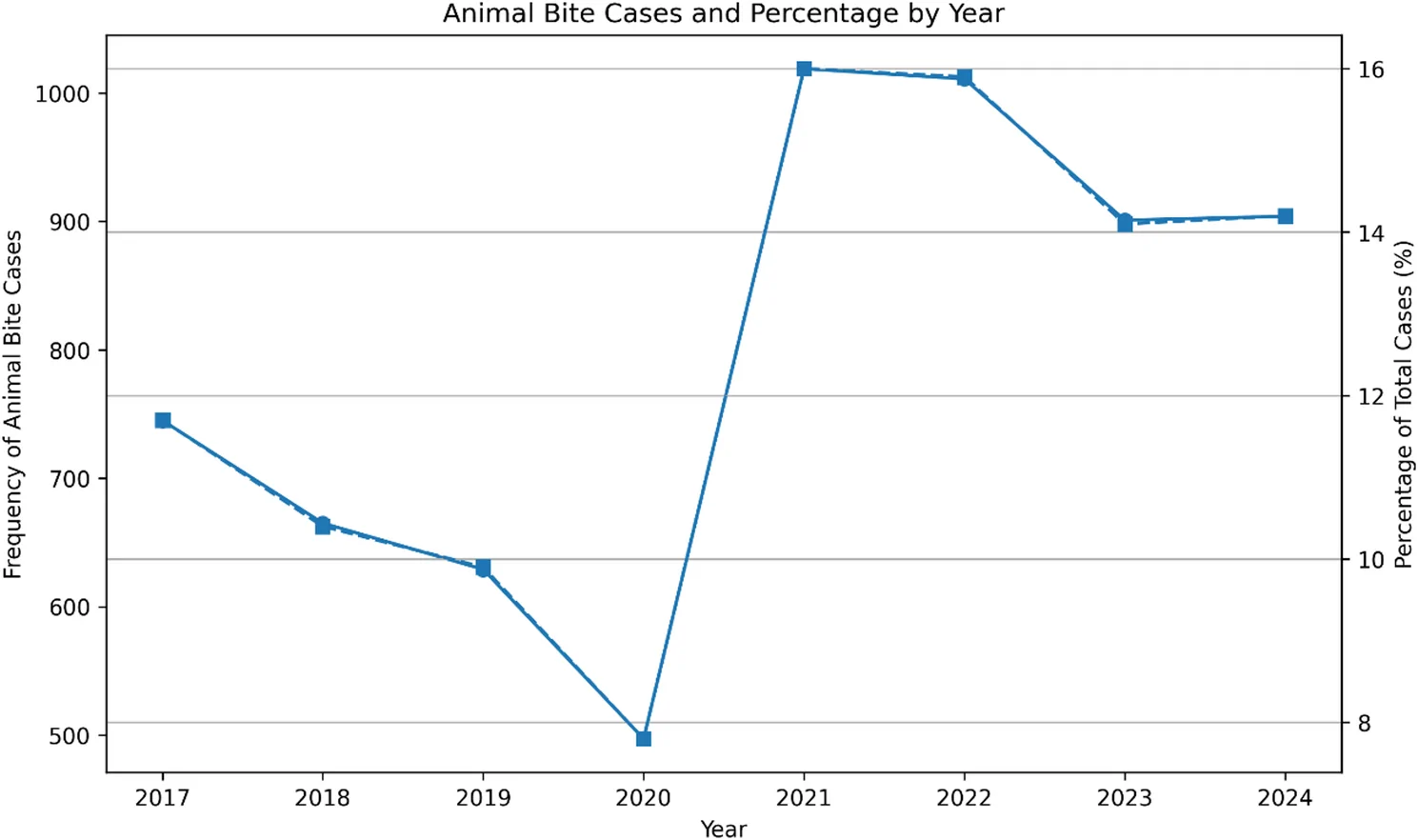
Animal bite trend report trends. In the frequency and percentage of animal bite cases from 2017 to 2024. The frequency is shown with a solid line and circular markers, while the percentage of total cases each year is shown with a dashed line and square markers

During the study period, a total of 6,371 animal bite cases were recorded. The average annual incidence rate of animal bites in Sarakhs County was 827.33 per 100,000 population. During the study, a total of 6,371 animal bite cases requiring medical intervention were recorded. The rate of reported animal bite cases increased over the years under study, from 745 cases (12%) in 2017 to 904 cases (14%) in 2024. Figure 2 and Table 1 illustrates the annual frequency and relative percentage of reported animal bite cases over the years 2017 to 2024. A noticeable decline in cases is observed from 2017 to 2020, followed by a sharp increase in 2021 and 2022. The trend then stabilizes in 2023 and 2024.

**Table 1:** Annual frequency and percentage distribution of animal bite cases in Sarakhs County, Iran, from 2017 to 2024

Year	Frequency of Animal Bite Cases	Percentage of Total Cases (%)
2017	745	11.7
2018	665	10.4
2019	629	9.9
2020	497	7.8
2021	1019	16.0
2022	1011	15.9
2023	901	14.1
2024	904	14.2
Total	6371	100

**Fig. 3:**
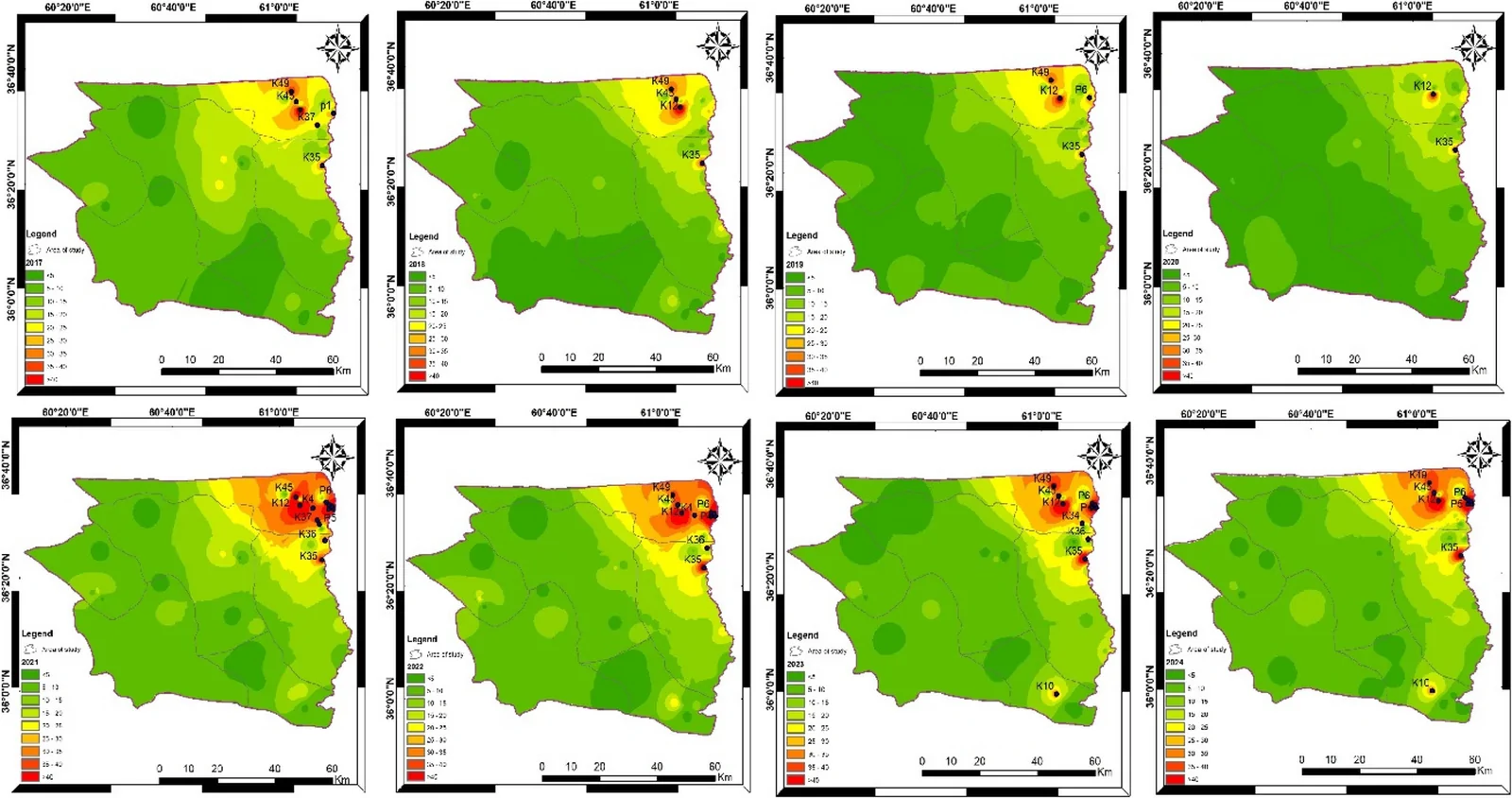
Zoning of Sarakhs County based on the severity of animal bites from 2017 to 2024

Between 2017 and 2024, the distribution of rabies cases in comprehensive health service centers in Sarakhs County shows that rural centers in the northeast region have the highest rates. As shown in Fig. 3, the red color highlights health centers where the number of animal bite cases exceeds 45 per year.

Table 2 summarizes the frequency and percentage distribution of animal bite incidents in Sarakhs County, categorized by gender, age group, occupation, and incident location (rural or urban). The data indicate a statistically significant association (*p* < 0.001) between these demographic and environmental factors and the frequency of animal bites. Males constitute the majority of bite victims, with the age group 20–49 years most affected. Students and housewives represent the highest occupational groups involved, and the vast majority of incidents occur in rural areas.

**Table 2:** Frequency distribution of animal bite cases in Sarakhs County by gender, age, occupation, and location of incident

Variable	Category	Frequency	Percentage (%)	Significance (*p*-value)
Gender	Male	5046	79.2	< 0.001
	Female	1325	20.8	
Age Group (years)	0–9	1087	17.1	< 0.001
	10–19	1579	24.8	
	20–49	2646	41.5	
	50 and above	1059	16.6	
Occupation	Self-employed	657	10.3	< 0.001
	Shepherd	189	3.0	
	Housewife	1007	15.8	
	Livestock breeder	491	7.7	
	Student	1790	28.1	
	Worker	554	8.7	
	Employee	287	4.5	
	Farmer	503	7.9	
	Child*	586	9.2	
	Others	307	4.8	
Location of Incident	Rural	5378	84.4	< 0.001
	Urban	993	15.6	

Table 3 presents the temporal distribution of animal bite incidents in Sarakhs County based on three dimensions: season, month, and time of day. The highest frequency of bites occurred during spring (27.2%), with a noticeable peak in Ordibehesht (April–May). The majority of bites occurred in the afternoon (13:00–18:00), accounting for 43.2% of total cases. Statistically significant relationships were observed between bite frequency and seasonal, monthly, and hourly variables (*p* < 0.001), suggesting a strong temporal pattern in the occurrence of animal bites.

**Table 3:** Distribution of animal bite cases in Sarakhs County by season, month, and time of day

Variable	Category	Frequency	Percentage (%)	Significance (*p*-value)
Season	Spring	1733	27.2	< 0.001
	Summer	1510	23.7	
	Autumn	1535	24.1	
	Winter	1593	25.0	
Month	Farvardin (Mar–Apr)	573	9.0	< 0.001
	Ordibehesht (Apr–May)	605	9.5	
	Khordad (May–Jun)	586	9.2	
	Tir (Jun–Jul)	535	8.4	
	Mordad (Jul–Aug)	490	7.7	
	Shahrivar (Aug–Sep)	459	7.2	
	Mehr (Sep–Oct)	471	7.4	
	Aban (Oct–Nov)	510	8.0	
	Azar (Nov–Dec)	561	8.8	
	Dey (Dec–Jan)	503	7.9	
	Bahman (Jan–Feb)	535	8.4	
	Esfand (Feb–Mar)	543	8.5	
Time of Day	00:00–06:00	198	3.1	< 0.001
	07:00–12:00	2249	35.3	
	13:00–18:00	2752	43.2	
	19:00–24:00	1172	18.4	

Table 4 provides detailed information on the types of animals responsible for bite incidents in Sarakhs County. The majority of bites were caused by dogs (90.1%), followed by cats (7.6%). Rare cases included bites from monkeys, bats, and weasels. Regarding animal ownership status, most of the animals were domestic (95%), and 65% of them had identifiable owners. Significant associations were observed between the frequency of animal bite cases and the type of animal (dog, cat, rodent, goat and sheep, donkey, wolf/fox/jackal, horse, cow/wild boar, bat, weasel, and monkey) as well as animal ownership status (domestic vs. wild) (*p* < 0.001).

**Table 4:** Distribution of animal bite cases in Sarakhs County by animal type and ownership

Variable	Category	Frequency	Percentage (%)	Significance (*p*-value)
Animal Type	Dog	5738	90.1	< 0.001
	Cat	484	7.6	
	Rodent (various types)	45	0.7	
	Goat and Sheep	38	0.6	
	Donkey	25	0.4	
	Wolf, Fox, Jackal	19	0.3	
	Horse	13	0.2	
	Cow and Wild Boar	3	0.05	
	Bat	2	0.03	
	Weasel	3	0.05	
	Monkey	1	0.02	
Animal Ownership	Domestic	6052	95.0	< 0.001
	Wild	319	5.0	
Animal Owner Status	Owned	4141	65.0	< 0.001
	Stray	2230	35.0	

Table 5 presents the characteristics of animal bite injuries in Sarakhs County, including the number of injuries sustained, the circumstances surrounding the bites, and the anatomical locations affected. Most individuals experienced a single injury (48.6%), and the most common cause of the bite was a sudden animal attack (72.8%). Lower limbs and buttocks were the most frequently injured areas (57.3%), followed by hands (17%). All examined variables demonstrated statistically significant associations with the frequency and nature of bite injuries (*p* < 0.001).

**Table 5:** Distribution of animal bite cases in Sarakhs County by injury characteristics and bite circumstances

Variable	Category	Frequency	Percentage (%)	Significance (*p*-value)
Number of Injuries	One	3042	47.8	< 0.001
	Two	1843	28.9	
	Three	771	12.1	
	More than Three	715	11.2	
Cause of animal bite	Teasing the animal	528	8.3	< 0.001
	Playing with the animal	449	7.1	
	Sudden attack	4639	72.8	
	Feeding the animal	313	4.9	
	Caring for the animal	377	5.9	
	Lab contact / rabid exposure	25	0.4	
	Other	45	0.7	
Injury Location	Head, face, neck	191	3.0	< 0.001
	Hand (fingers to wrist)	1093	17.2	
	Forearm, arm, shoulder	350	5.5	
	Chest, abdomen, back	293	4.6	
	Upper limb	816	12.8	
	Lower limb, buttocks	3650	57.3	
	Genital/mucosal/perineal	8	0.1	
	Eye, eyelid, nose, mouth	1	0.0	

## Discussion

Investigating the factors associated with animal bites, particularly demographic characteristics such as age, gender, and occupation, as well as environmental conditions, is essential for prevention and control strategies. Understanding these patterns can support the development of effective educational and preventive interventions.

The findings of this study showed a fluctuation in the trend of animal bites in Sarakhs County over the study period, with an increasing pattern observed in recent years. Similar increasing trends have been reported in other regional and national studies, suggesting a growing public health concern related to animal exposure and rabies risk.

Several factors may explain this increase. Disruptions caused by the COVID-19 pandemic may have reduced routine animal control and surveillance activities. In addition, increased movement between rural and urban areas after the lifting of quarantine restrictions, along with possible growth in stray dog populations and reduced veterinary public health interventions, may have contributed to higher exposure rates. Moreover, improved reporting systems and increased public awareness may also have led to better case registration.

In contrast, the earlier decline observed between 2017 and 2020 may be attributed to enhanced public health education, vaccination campaigns targeting livestock and stray dogs, and coordinated control measures implemented by veterinary and municipal authorities.

Conversely, the increase after 2020 may signal a weakness in the sustainability of these measures. It is crucial to continue community-based preventive actions, strengthen livestock vaccination efforts, and implement targeted control of stray animal populations to reduce the number of cases again.

Analysis of gender and age distribution reveals that a significant majority of bite victims are male, with men accounting for 75.48% to 83.4% of cases. Analysis of gender and age distribution in the present study revealed that a significant majority of bite victims were male, accounting for 75.48% to 83.4% of cases. Regarding age distribution, younger individuals were more frequently affected, with the highest incidence observed in the 5–24 or 21–30 age groups.

Consistent with previous studies, rural areas have been reported to experience higher rates of animal bite incidents, and similar patterns of higher vulnerability among children and young adults have been documented in the literature [1, 16]. Occupational risk is elevated for students, farmers, and ranchers. Seasonal variations also play a role, as bite rates are higher in spring and summer [1, 12].This trend may suggest that gender influences the risk of animal interactions, as men tend to have more exposure to animals in both occupational and recreational settings. The most affected age group is 20 to 49 years, which is concerning since this population usually engages in outdoor activities or jobs that may increase their risk of bites. Additionally, the distribution of bite victims by occupation illustrates that students and housewives are the most impacted groups. This vulnerability may stem from students’ frequent interactions with animals during recreational activities and housewives’ responsibilities for animal care at home. Most animal bite incidents occur in rural areas, highlighting that the rural environment, which is closer to animals and has fewer medical resources, significantly contributes to the frequency of these events. The higher incidence rate in these regions may also relate to cultural factors that normalize interactions with animals in agricultural societies.

The results of this study reveal a distinct temporal pattern in the occurrence of animal bites in Sarakhs County. The highest number of cases was recorded in spring, particularly in May, with the most frequent occurrences noted in the afternoon hours (between 1 PM and 6 PM), accounting for over one-third of all cases. Animal bites exhibit seasonal patterns, with increased rates observed in both summer and winter months across different regions. Research conducted in Iran and Turkey found peaks in bite cases during June and August،, while studies in India indicated that the highest number of bites occurs in winter, followed by the monsoon or summer seasons [17]. These seasonal patterns can often be attributed to heightened agricultural and grazing activities during the spring and summer, which lead to greater human and animal interaction, increased exposure, and favorable weather conditions that allow for more animal movement [18]. The rise in bite cases during the afternoon can also be connected to people gathering outdoors and encountering dogs or other animals at that time. Notably, this pattern has remained consistent over recent years, showing no unexpected fluctuations. This stability suggests a predictable interplay of behavioral and environmental factors in the community. Such consistency enables the development of focused educational programs, including seasonal outreach initiatives or time-specific interventions, such as educational efforts in May or awareness campaigns during the afternoon and evening.

According to the findings of this study, most animal bites in Sarakhs County were caused by dogs, followed by cats. Other animals, such as rats, donkeys, foxes, and bats, accounted for a very small percentage of the incidents. Notably, 95% of the attacking animals were domesticated and had known owners. All of these factors were found to be statistically significant in relation to the occurrence of animal bites. Studies from Iran indicate consistent patterns in the epidemiology of animal bites, with dogs being the primary source, responsible for 77.8% to 91.4% of case [1, 12, 16]. Dog bites present a significant public health concern, as human behavior plays a crucial role in these incidents; most bites occur during play or interaction with the animals [19]. To reduce the incidence of dog bites and their associated public health risks, it is essential to improve public health education, promote responsible dog ownership, and ensure adequate vaccination coverage against rabies, the most important zoonotic disease associated with dog bites, as well as other canine infectious diseases [20]. Dog ownership itself is a considerable risk factor for bites [21]. In many rural areas of Iran, including Sarakhs County, dogs are often kept for guarding livestock and may roam freely in yards, having close contact with humans, especially children. In contrast to urban dogs, which tend to be more controlled and vaccinated, rural dogs may be untrained or aggressive. The high percentage of bites from “owned” animals (65%) suggests that the issue is not solely related to strays but stems largely from inadequate training for pet owners and a lack of familiarity with safe animal handling practices. Given the climate and geography of the area, the likelihood of direct contact with wild animals, such as bats, foxes, jackals, or raccoons, is much lower than with domestic animals. In some cases, people approach domestic animals like dogs and cats for companionship or work-related purposes (such as playing, feeding, or guarding), which increases the likelihood of bites.

The findings of this study revealed that most animal bites resulted in a single area of injury, with sudden attacks by animals being the primary cause of these incidents. Additionally, activities such as playing with, teasing, feeding, or caring for the animal were also identified as contributing factors. This indicates that a significant number of bites occur during everyday interactions with domestic or semi-domestic animals. The most common sites of injury were the lower extremities, particularly the legs and buttocks. This is predictable, as initial contact with an animal, especially a dog, typically occurs at a lower level. However, there were also a few cases involving injuries to the head, face, and neck. These injuries are clinically and epidemiologically significant because they increase the risk of rabies virus transmission and require immediate medical attention. Overall, dog bites are complex incidents influenced by various factors, including human behavior, dog characteristics, and environmental contexts.

Common causes of dog bites include interactions or attempts to interact with dogs, such as petting, playing with, or touching them [22, 23]. Victims often know the dog, and many of these incidents occur indoors. Males and children, particularly those aged 5 to 9 years, are more frequently affected [23, 24]. The location of the bite varies, but studies indicate that the lower extremities are the most common sites [1]. Children are more likely to sustain injuries to the head and neck, while adults typically suffer bites to the extremities [24]. Human error, such as interfering with restrained dogs or engaging in fights between dogs, significantly contributes to bite incidents [25]. The high number of cases involving only one affected area suggests that many dog bites are resolved quickly. However, 22% of cases with three or more lesions in the affected area may indicate the severity of the attack or the inability to control the attacking animal. This highlights the need for improved prevention programs and clinical care. Injuries to the hands are particularly common, as this area is often involved in close interactions, such as playing, feeding, or caring for the animal, especially among children and adolescents.

These findings underscore the importance of targeted prevention strategies and educational programs, as well as the need for public health interventions to reduce the incidence of animal bites, particularly in rural areas. Educational campaigns focusing on safe interactions with animals, proper handling techniques, and awareness of the risks associated with animal bites could be especially beneficial for the high-risk groups identified in this study.

One of the main limitations of this study was the incomplete information recorded in the files of the affected individuals. Efforts were made to fill in the gaps by consulting relevant experts and contacting the individuals when possible. Another potential limitation was the risk of incorrect data transfer from the files to the checklist. To address this, the researcher made sure to verify all information at least twice.

## Conclusion

The findings of this study indicate that health and veterinary professionals need to be particularly vigilant in the spring, especially in May. It is essential to plan information and education sessions during late morning and early afternoon hours. Interventions should focus on strengthening animal vaccination efforts, training on appropriate interactions with animals, and improving access to medical centers. Public education should emphasize the responsibilities of pet owners and adhere to safe practices, such as ensuring pets wear collars, providing proper training, and monitoring children’s interactions with dogs. While controlling stray dog populations is important, prioritizing education and vaccination for owned animals may have a more significant impact on reducing animal bites. This reinforces the need for well-designed educational programs that aim to prevent high-risk encounters with animals, effectively manage pet dogs, and teach safe behaviors when interacting with animals. More emphasis should be placed on counseling and educational programs that promote awareness and responsible conduct, particularly when walking, biking, or interacting with animals in rural areas. Additionally, it is recommended to provide education on recognizing animal behavior for both children and adults when they encounter animals closely.

## Data Availability

All data generated or analyzed during this study are included in this published article.
